# Antimicrobial Activity of Xoconostle Pears (*Opuntia matudae*) against *Escherichia coli* O157:H7 in Laboratory Medium

**DOI:** 10.1155/2012/368472

**Published:** 2012-08-15

**Authors:** Saeed A. Hayek, Salam A. Ibrahim

**Affiliations:** Food Microbiology and Biotechnology Laboratory, North Carolina Agricultural and Technical State University, Greensboro, NC 27411, USA

## Abstract

The objective of this study was to investigate the antimicrobial activity of xoconostle pears (*Opuntia matudae*) against *Escherichia coli* O157:H7. Xoconostle pears were sliced, blended, and centrifuged. The supernatant was then filtered using a 0.45 **μ**m filter to obtain direct extract. Direct extract of xoconostle pears was tested against four strains of *E. coli* O157:H7 in brain heart infusion (BHI) laboratory medium using growth over time and agar well diffusion assays. Our results showed that direct extract of xoconostle pears had a significant (*P* < 0.05) inhibitory effect at 4, 6, and 8% (v/v) concentrations and complete inhibitory effect at 10% (v/v) during 8 h of incubation at 37°C. Minimum inhibitory volume (MIV) was 400 **μ**L mL^−1^ (v/v) and minimum lethal volume (MLV) was 650 **μ**L mL^−1^ (v/v). The inhibitory effect of xoconostle pears found to be concentration dependent and not strain dependent. Thus, xoconostle pears extract has the potential to inhibit the growth of *E. coli* O157:H7 and could provide a natural means of controlling pathogenic contamination, thereby mitigating food safety risks.

## 1. Introduction

Foodborne pathogens are major concern to consumers, food industry, and food regulatory agencies. The yearly cost of foodborne illnesses in the United States as reported in 2010 was about $152 billion including $993 million caused by *Escherichia coli* O157:H7 in healthcare, workplace, and other economic losses [1]. *E. coli* O157:H7 is one of the most important foodborne pathogens in the United States, having been first identified in 1982. From 1982 to 2002, *E. coli* O157:H7 caused 73,000 illnesses annually in the United States including 8,598 infection cases and 40 deaths [2]. *E. coli* O157:H7 causes severe gastrointestinal diseases such as bloody diarrhea haemorrhagic colitis, haemolytic uremic syndrome, and traveler's diarrhea [3, 4]. Therefore, the control and prevention of *E. coli *O157:H7 in food products is an area that is receiving worldwide attention.

Many food preservation techniques have been developed to control and prevent foodborne pathogens including antimicrobials. Most antimicrobials used in the food industry are chemical preservatives [3]. Even though chemical preservatives have been approved by many countries and used for years, they are considered by most consumers to be unhealthy [3, 5]. Therefore, natural antimicrobials have become increasingly important to the food industry in order to meet the consumers demands [5]. Natural antimicrobials can be found in a variety of plants including herbs, spices, fruits, vegetables, and tropical plants [5, 6]. Plants contain an array of natural compounds with many medicinal benefits and provide about 50% of current pharmaceuticals [6]. Even though, several reports have demonstrated the efficacy of using natural ingredients [7–10] and plant extractions [11–16] to control the growth of foodborne pathogens, no plants are currently used as antimicrobials [6]. Thus, food industries are very motivated to replace chemical preservatives with natural antimicrobials. 

Xoconostle pears (*Opuntia matudae*) have attracted the attention of researchers around the world due to this particular pear's strong anticancer, antidiabetic, and antioxidant characteristics [17–19]. Xoconostle pears are rich source of soluble phenolics, ascorbic acid, and betalains compared to most common fruits and vegetables [19]. Soluble phenolics, betalains, and ascorbic acid have already shown effective antimicrobial activity in many studies [6, 8, 10–12, 14–16]. Thus xoconostle pears have great potential as natural antimicrobial. To the best of our knowledge, there is no information in the literature on the antimicrobial activity of xoconostle pears against any pathogenic bacteria including *E. coli *O157:H7. Therefore, the objective of this study was to examine the antimicrobial activity of xoconostle pears direct extract against *E. coli *O157:H7 in brain heart infusion (BHI) laboratory medium. 

## 2. Materials and Methods

### 2.1. Bacterial Culture Activation and Preparation

Four strains of *E. coli* O157:H7, F4546 (alfalfa sprout isolate), H1730 (lettuce isolate), 43895 (beef isolate), and 944 (salami isolate) were used in this study. These *E. coli* O157:H7 strains were selected from different isolating sources and have been associated with several outbreaks. The* E. coli* O157:H7 strains were supplied by Dr. S. S. Summer, Department of Food Science and Technology at Virginia Tech, and stored at −80°C freezer stock storage of our laboratory. Immunoblot using Protran nitrocellulose membranes (BA85, Whatman, Schleicher and Schuell, Sanford, ME) was performed to identify the *E. coli* O157:H7 strains [20]. A confirmation step usingpolymerase chain reaction(PCR) assay was also conducted to identify the serogroup of *E. coli* O157:H7 strains [21]. The strains were activated in BHI (Becton Dickinson, Sparks, MD, USA) broth by transferring 100 *μ*L from the stored culture to 10 mL BHI broth and incubating at 37°C for 24 h. Activated strains were stored in a refrigerator at 4°C. Prior to each experimental replication, each individual bacterial strain was streaked on BHI agar and incubated for 24 h at 37°C. One isolated colony was transferred to 10 mL BHI broth, and incubated at 37°C for next day use.

### 2.2. Xoconostle Extract Preparation

Xoconostle pears were obtained from a local grocery market in Greensboro, NC. For each experiment replication, 450 g of fresh xoconostle pears were rinsed under running tap water, blotted with paper towel, sliced into small pieces, and blended in a kitchen blender for 4 min. This preparation was placed in 50 mL tubes and centrifuged at 7800 × g for 10 min using Thermo Scientific* Sorvall RC 6 Plus Centrifuge (Thermo Scientific Co., Asheville, NC, USA). The supernatant was filtered using a 0.45 *μ*m Nalgene filter (Nalge Nunc International Corp, Rochester, NY, USA) to collect the xoconostle direct extract which was stored at 4°C until used within 12–16 h. 

### 2.3. Bacterial Enumeration

Bacterial populations were determined by plating onto BHI agar. In this procedure, samples were individually diluted into serial of 9 mL 0.1% peptone water solution (Bacto peptone, Becton Dickinson, Sparks, MD, USA); (pH 7.25 ± 0.08); then 100 *μ*L of appropriate dilutions were surface plated onto triplicates BHI agar plates and incubated at 37°C for 24 h. Plates with colonies ranging between 30–300 were considered for colony counting to determine the bacterial populations [3].

### 2.4. Growth Over Time Assay

Growth over time assay was employed following the instructions by Marwan and Nagel [22] and Parish and Carroll [23] with slight modifications. Overnight activated bacterial strains were individually diluted into serial of 9 mL 0.1% peptone water solution to obtain a bacterial population of approximately 4 log CFU mL^−1^. For each individual strain, batches of sterilized tubes containing 9 mL BHI broth were mixed with xoconostle extract to obtain different concentrations (4, 6, 8, and 10% v/v) and inoculated with 1 mL of the previously diluted individual bacterial strains. Control samples without xoconostle extract for each individual bacterial strain and blank samples without bacterial inoculation for each treatment level were included. The initial bacterial populations for each strain were approximately 3 log CFU mL^−1^ and that was determined using the bacterial enumeration method previously described. Samples were incubated with shaking at 37°C for 8 h, and bacterial growth was monitored by measuring the optical density (O.D. 610 nm) at 2 h interval using Thermo Scientific Genesys 10S UV-Vis spectrophotometer (Thermo Fisher Scientific Co., Madison, WI, USA). The final bacterial populations were determined at the end of the incubation period. 

### 2.5. Agar Well Diffusion Assay

Agar well diffusion assay described by Hugo and Russel [24] and Ibrahim and others [16] with slight modifications was employed. Individual strain was grown overnight then serially diluted into 9 mL 0.1% peptone water solution to obtain bacterial populations of 6 log CFU mL^−1^approximately. Diluted strains at 10 mL each were mixed in a sterilized container. BHI agar at 500 mL with 0.2% of Tween 80 was prepared and sterilized at 121°C for 15 min. Prepared BHI agar was placed in a water bath at 48°C and allowed to cool down then inoculated with 20 mL of previously mixed culture to achieve a bacterial population of 4-5 log CFU mL^−1^. Inoculated BHI agar was poured into Petri dishes (15 × 100 mm^2^) at approximately 50 mL each and allowed to solidify in 30 min under biohazard cabinet. A sterile cork borer (8.0 mm diameter) was used to punch wells in the inoculated agar. The agar plugs were removed using a sterilized wire loop. Xoconostle extract at different volumes (200–1000 *μ*L with 25 *μ*L unit increase) were adjusted with sterilized distilled water to 1 mL to obtain different concentrations (v/v) and poured into the wells to the top. Plates were kept under a biohazard cabinet for 30 min for prediffusion to occur, incubated at 37°C for 12 h, and then examined for the development of clear inhibitory zone. Observed inhibitory zones were measured to the nearest 0.1 mm and reported after subtracting the well diameter from the observed zone diameter. Minimum inhibitory volume (MIV) was determined at this point. Incubation of the plates was continued for three days to determine the minimum lethal volume (MLV). MIV was defined as the lowest volume concentration that caused significant inhibitory effect during 12 h of incubation at 37°C and MLV was defined as the lowest volume concentration that showed significant inhibitory effect after three days of incubation [16, 25]. Inhibitory zone at 3 mm or larger was considered significant [25, 26].

### 2.6. Statistical Analysis

Each experimental test was conducted three times to determine the effect of xoconostle pears on the growth of *E. coli* O157:H7. Mean values and standard deviations were calculated from the triplicate samples. Statistical analysis system (SAS) [27] version 9.2 was used to determine significant antimicrobial activity at different concentrations of xoconostle extract and significant differences among strains at the same concentration of xoconostle extract using the data means by a factorial analysis of variance of triplicate samples at a significant level of *P* < 0.05. 

## 3. Results

**Figure 1:**
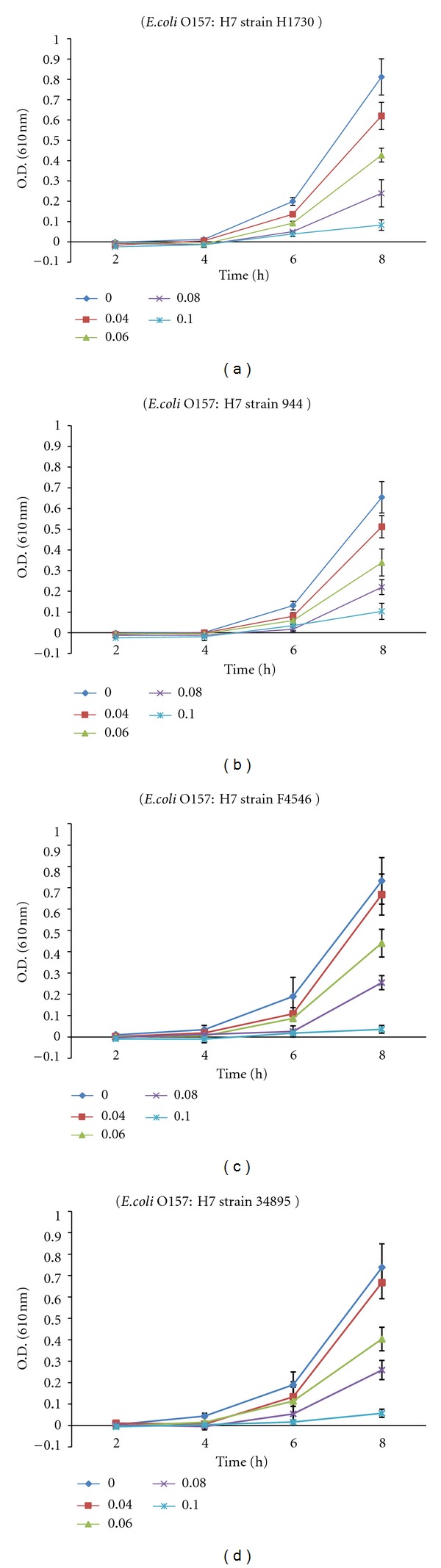
Bacterial growth curve of *E. coli* O157:H7 strains in the present of xoconostle extract at different concentrations % (v/v) inBHI brothduring 8 hof incubation at 37°C measured by turbidity (optical density 610 nm). Data points are the average of 3 replicates with standard error.

Figure 1 shows the growth of *E. coli* O157:H7 in BHI broth treated with different volumes of xoconostle extract during 8 h of incubation at 37°C. In control samples, optical density readings reached absorbance in the range of 0.654–0.812 (O.D. 610 nm). When* E. coli* O157:H7 strains were grown in BHI broth treated with 4, 6, 8, and 10% (v/v) xoconostle extract, optical density readings reached ranges of 0.512–0.668, 0.339–0.440, 0.220–0.259, and 0.036–0.103 (O.D. 610 nm) respectively. An optical density reading of 0.1 (O. D. 610 nm) or less was previously defined as the division between visual growth and no growth [23, 25]. Thus, xoconostle extract at 4, 6, and 8% (v/v) concentrations was able to slow down the growth of *E. coli* O157:H7 strains whereas 10% was enough to cause no growth.  Table 1 shows the final population of *E. coli* O157:H7 strains grown in BHI broth treated with different concentrations (v/v) of xoconostle extract after 8 h of incubation at 37°C. In control samples, *E. coli* O157:H7 continued to grow and reached the stationary phase. The additions of xoconostle extract at 4, 6, 8, and 10% (v/v) caused significant (*P* < 0.05) reductions in *E. coli* O157:H7 populations at averages of 0.99 ± 0.17, 2.23 ± 0.35, 3.66 ± 0.22, and 5.78 ± 0.41 log CFU mL^−1^, respectively. Samples treated with 10% (v/v) xoconostle extract caused final bacterial populations to remain within the initial count range (about 3 logs CFU mL^−1^). These results indicate that xoconostle pears have a significant inhibitory effect on *E. coli* O157:H7, and 10% (v/v) concentration of xoconostle extract is required to achieve complete growth inhibition.

Table 2 shows the inhibitory zones (with 100 *μ*L unit increase) that were formed around the wells after 12 h of incubation at 37°C. Bacterial growth developed a greenish cloud all over the agar whereas distinguishable clear zone remained around the well. The lowest concentration that shows a clear inhibitory zone was 275 *μ*L mL^−1^ (v/v) with an average of 1.0 ± 0.2 mm. MIV was recorded for a significant inhibitory effect at 400 *μ*L mL^−1^ (v/v) with an average of 2.9 ± 0.2 mm. When xoconostle extract without dilution was transferred to the well, the average inhibitory zone reached 9.8 ± 1.01 mm. After three days of incubation, MLV was recorded for a significant inhibitory effect at 650 *μ*L mL^−1^ (v/v) with an average of 2.8 ± 0.25 mm. These data support the growth over time assay results indicating that xoconostle pears had significant inhibitory effect on *E. coli* O157:H7. 

## 4. Discussion

An increasing consumer demand for food products that are minimally processed and contain natural ingredients has been noticed recently.This demand has resulted in an effort by the food industry to search for natural antimicrobials. In the present work, the antimicrobial activity of xoconostle pears was studied using growth over time and well diffusion assays. Both assays are common for studying the antimicrobial activity in food microbiology [14, 23, 25, 26] and our laboratory has used both assays with consistent results [7, 9, 10, 13, 16]. The growth over time and well diffusion assays are practical, simple, and could be used for direct screening of direct extracts from fruits and vegetables. Direct extract of xoconostle pears was obtained mechanically without any chemical, heating, or concentration processing. Direct extraction is a simple and safe procedure that can avoid any possible alteration to or destruction of the native structure of the active ingredients. Common extraction procedures include chemical or heating treatments could alter the active ingredients total content, functionality, natural characteristics, or could produce unsafe compounds [28, 29]. In addition, direct extracts from fruits or vegetables can be applied to food products in a safe manner. 

Our results showed that xoconostle pears have significant inhibitory effect on the growth of *E. coli* O157:H7. Figure 1 and Table 1 show that the increase in xoconostle extract concentration associated with a slower growth rate and more bacterial populations reductions, respectively. Table 2 shows that the gradual increase in the inhibitory zone with respect to the increase in xoconostle extract concentration. These results indicate that the inhibitory effect of xoconostle extract is concentration dependent. On the other hand, Table 1 shows no significant (*P* > 0.05) differences in the final bacterial populations among *E. coli* O157:H7 strains grown at the same concentration of xoconostle extract except for *E. coli* O157:H7 43895. However, different *E. coli* O157:H7 strains may grow at different growth rates [30] which may explain the difference in the final bacterial population of *E. coli* O157:H7 43895. Thus, xoconostle pears have the same level of antimicrobial activity on different* E. coli* O157:H7 strains. Therefore, the inhibitory effect of xoconostle pears is concentration dependent and not strains dependent.

The antimicrobial activity of xoconostle pears can thus be accounted for several active compounds including phenolics, ascorbic acid, and betalains. Xoconostle pears have a combination of phenolic compounds including gallic, vanillic, 4-hydroxybenzoic acids, catechin, epicatechin, and vanillin [19]. Even though the exact antimicrobial mechanism of phenolic compounds is not clear, phenolic compounds are commonly known for their antimicrobial effects [6]. The ability of phenolic compounds to alter microbial cell permeability, thereby permitting the loss of macromolecules from the cell interior, could help explain some of the antimicrobial activities [11]. Another explanation might be that phenolic compounds interfere with membrane function and interact with membrane proteins, causing deformation in structure and functionality [11]. A combination of phenolic compounds can provide synergistic antimicrobial effects and can contribute to better antimicrobial reaction as compare to the reaction of an individual compound [15]. The nature of xoconostle pears containing several phenolic compounds may contribute to strong antimicrobial activity. Xoconostle pears are also rich in ascorbic acid and have higher amount of ascorbic acid than most common fruits and vegetables such as raspberry, red plum, green grape, pear, apple, peach, banana, onion, spinach, green cabbage, pea, cauliflower, lettuce, and tomato [19]. Ascorbic acid is well characterized as a reducing agent with free chemical radicals in chemical and biological systems [8, 14]. In addition, ascorbic acid has the ability to absorb oxygen which might provide a barrier against available oxygen required for *E. coli* O157:H7 [8]. However, ascorbic acid alone has a weaker inhibitory effect compared to the synergetic effect of ascorbic acid and phenolic compounds [8, 10].Furthermore, xoconostle pears are rich in betalains that are well documented for excellent antiradical and antioxidant activity [31, 32]. Betalains can also act as modulators of adhesive molecule expression in endothelial cells [31]. Betalains have metal chelating activities in which they can chelate the cell's indispensable inner cations Ca^2+^, Fe^2+^, and Mg^2+^ [12]. These characteristics of betalains have received increased attention suggesting antiviral and antimicrobial activities [12, 32]. The strong antioxidant properties of betalains might provide an additional barrier with ascorbic acid against available oxygen required for *E. coli* O157:H7. The presence of a combination of soluble phenolics with ascorbic acid and betalains might introduce a strong synergetic antimicrobial effect. Therefore, it would be possible to suggest that antimicrobial activity of xoconostle pears is due to the synergistic effect of these active compounds.

Based on the results of these experiments, we suggest that it is possible to use direct extract from xoconostle pears as natural antimicrobial agent against *E. coli* O157:H7. Since the inhibitory effect of xoconostle pears was found to be concentration dependent and not strain dependent, the effective concentration must be determined for successful industrial applications. These findings may thus lead to more attention to the antimicrobial activity of xoconostle pears against other pathogens. Further work is needed to determine the impact of xoconostle pears on* E. coli* O157:H7 in various food applications. Future work in our laboratory will be conducted to determine the antimicrobial activity of xoconostle pears against other pathogenic bacteria including *Salmonella* and *Listeria monocytogenes* and to be tested in food model. 

## Figures and Tables

**Table 1:** Final population of *E. coli* O157:H7 strains in BHI broth in the presence of xoconostle extract at different concentrations % (v/v) after 8 h of incubations at 37^°^C. Data represent the average of three replicates with standard error.

xoconostle extract Concentration % (v/v)	Final population (Log CFU/mL) of *E. coli* O157:H7 strains			
	H1730	944	F4546	43895
0 (control)	9.19^aA^ ± 0.16	8.66^aA^ ± 0.12	9.12^aA^ ± 0.20	9.31^aA^ ± 0.13
4	8.09^bA^ ± 0.09	7.92^bA^ ± 0.14	8.08^bA^ ± 0.17	8.25^bA^ ± 0.15
6	6.79^cA^ ± 0.19	6.75^cA^ ± 0.14	6.78^cA^ ± 0.17	7.04^cA^ ± 0.15
8	5.32^dA^ ± 0.15	5.16^dA^ ± 0.18	5.32^dA^ ± 0.11	5.90^dA^ ± 0.14
10	3.08^eA^ ± 0.16	2.98^eA^ ± 0.16	3.07^eA^ ± 0.12	4.02^eB^ ± 0.09

*Averages with different lower case letters in the same column are significantly different (*P* < 0.05).

*Averages with different upper case letters in the same row are significantly different (*P* < 0.05).

**Table 2:** Inhibitory zones in BHI agar inoculated with mixture of *E. coli* O157:H7 strains that formed around the wells due to the present of xoconostle extract at different concentrations (v/v) after 12 h of incubation at 37^°^C. Concentrations are in *μ*L adjusted to 1 mL by distilled water and inhibitory zone = diameter of the zone −8 mm (diameter of the well). Data represent the average of three replicates with standard error.

Concentration (*μ*L/mL)	Inhibitory zone (mm)
200	0
300	1.4 ± 0.3
400	2.9 ± 0.2
500	4.3 ± 0.45
600	5.7 ± 0.3
700	6.6 ± 0.36
800	7.7 ± 0.35
900	9.0 ± 0.45
1000	9.8 ± 1.01
